# Analysis of the Prevalence of Alcohol and Psychoactive Substances Among Drivers in the Material from the Department of Forensic Medicine at the Medical University of Bialystok in Poland †

**DOI:** 10.3390/toxics13110960

**Published:** 2025-11-06

**Authors:** Michal Szeremeta, Julia Janica, Gabriela Jurkiewicz, Marta Galicka, Julia Koścień, Julia Więcko, Jakub Perkowski, Michal Krzysztof Jeleniewski, Karol Siemieniuk, Anna Niemcunowicz-Janica

**Affiliations:** 1 Department of Forensic Medicine, Medical University of Bialystok, Waszyngtona St. 13, 15-269 Bialystok, Poland; anna.niemcunowicz-janica@umb.edu.pl; 2 Forensic Medicine Students’ Association, Department of Forensic Medicine, Medical University of Bialystok, Waszyngtona St. 13, 15-269 Bialystok, Poland; gabrielajurkiewicz99@gmail.com (G.J.); 39859@student.umb.edu.pl (M.G.); 39886@student.umb.edu.pl (J.K.); 39972@student.umb.edu.pl (J.W.); 39927@student.umb.edu.pl (J.P.); 39874@student.umb.edu.pl (M.K.J.); 39945@student.umb.edu.pl (K.S.)

**Keywords:** driving under the influence, toxicological analysis, road accident, roadside survey

## Abstract

In recent years, the issue of drivers under the influence of medications and psychoactive substances as a cause of road accidents has gained increasing importance. This study aimed to assess the prevalence and blood concentration ranges of alcohol and psychoactive substances among drivers in northeastern Poland between 2013 and 2024. To determine the prevalence of medications and psychoactive substances in drivers’ blood, data were collected from 266 blood samples obtained from drivers (251 men and 15 women). Among these, 79 drivers died immediately, 61 drivers survived the accident, and 126 drivers were stopped for roadside checks. The presence of the studied substances was confirmed using gas chromatography combined with mass spectrometry detection (GC-MS) and liquid chromatography combined with mass spectrometry detection (LC-MS). Blood alcohol content was measured using headspace gas chromatography with a flame ionisation detector (HS-GC-FID). Psychoactive substances were detected in 152 of the 266 samples. Drivers testing positive for medications and psychoactive substances were most frequently stopped during roadside controls—67.46%. Among the total positive cases, psychoactive substances used alone or in combination included THC—46.3% (range 0.2–20 ng/mL), alcohol—26.8% (range 0.1–4.1‰), amphetamines—20.7% (range 15–2997 ng/mL), opiates—4.3% (morphine 66.0 ng/mL; methadone 174.0 ng/mL; ranges: tramadol 15.0–600.0 ng/mL; fentanyl 45.0–100.0 ng/mL), benzodiazepines—9.8% (ranges: diazepam 55.0–480.0 ng/mL; midazolam 17.0–1200.0 ng/mL; clonazepam 21.0–36.0 ng/mL), stimulants—6.10% (ranges: amphetamine 15.0–2997.0 ng/mL; cocaine 4.0–30.0 ng/mL; benzoylecgonine 38.0–602.0 ng/mL; PMMA 45.0–360.0 ng/mL; MDMA 20.0–75.0 ng/mL; mephedrone 37.5 ng/mL; alfa-PVP 120 ng/mL), psychotropic drugs—3.1% (carbamazepine 8.0–2100.0 ng/mL; zolpidem 233.0 ng/mL; citalopram 320.0 ng/mL; opipramol 220 ng/mL). The most commonly used substance among car and motorcycle drivers was THC (37.7% of car drivers and 60% of motorcyclists). Among operators of other types of vehicles, alcohol was the most frequently detected substance, present in 35% of cases. The majority of drivers (81.1%) were under the influence of a single substance. Among the drivers, 7.3% consumed alcohol in combination with at least one other substance, and 11.6% used two or more substances excluding alcohol. Among the psychoactive substances most frequently used alone or in combination with others, THC was predominant. Roadside testing, based on effects similar to alcohol intoxication, was mainly conducted on male drivers.

## 1. Introduction

In recent years, driving under the influence of alcohol (DUIA) and/or driving under the influence of drugs (DUID) has become a more serious issue. Driving under the influence of alcohol is known to increase the risk of road accidents, with the relative risk rising as blood alcohol concentration increases [1]. Less is known about the effects of medications or illicit drugs [2], and there is debate about a clear link between drug levels and accident risk [3]. Certain substances, such as alcohol, amphetamines, cannabis, opioids, or cocaine, can impair visual, cognitive, or motor skills necessary for safe driving [4]. For example, the legalisation of recreational use of cannabis for people over 21 years of age in the United States of America [5] led to an increase in road accidents with severe and fatal injuries being recorded, and cannabis was detected in 25.1% of the biological samples of the drivers [6,7]. Various countries have developed their own strategies to tackle the issue. Norway and Sweden have adopted a zero-tolerance policy for driving under the influence of psychoactive substances, which has reduced positive cases [8]. In Poland, due to the Polish Criminal Code (Art. 178a), anyone who, while in a state of insobriety or under the influence of an intoxicant, drives a motor vehicle on land, water or in the air is liable to a fine, with the restriction of liberty or imprisonment for up to three years. This is why “driving under the influence” in Poland is prohibited. In contrast, other countries have set threshold limits.

Alcohol is the only substance with official international cut-off values and standardised analytical methods [4]. In Poland, the “state of sobriety” ends once a driver’s blood alcohol content (BAC) exceeds 0.2‰. At this point, the driver is classified as being in a “state after the use of alcohol”. Driving with a BAC between 0.2‰ and 0.5‰ is a misdemeanour. Exceeding 0.5‰ is a criminal offence.

Creating similar procedures for psychoactive substances is complex. The rapid molecular evolution of many illicit drugs makes it difficult. New psychoactive substances (NPS) clearly illustrate this challenge. Testing for these substances in both clinical and laboratory settings is demanding. It requires highly specialised methods and expertise, which are not widely available [9].

The number of road accidents involving driving under the influence of alcohol has been steadily decreasing since 2009. Over the past twelve years in Poland, the number of accidents has decreased from 4028 in 2013 [10] to 1944 in 2024 [11]. Similarly, police statistics indicate that the number of people caught “driving under the influence” fell from 128,064 in 2013 to 49,647 nationwide in 2023, and from 4740 to 1838 in Podlasie Province [12].

Despite the decline in alcohol and psychoactive substance-related accidents, our study examines the presence and concentrations of these substances in the blood of three groups: drivers who were stopped for roadside surveys, drivers who survived accidents, and drivers involved in fatal traffic accidents. This study aimed to assess the prevalence and blood concentration ranges of alcohol and psychoactive substances among drivers in northeastern Poland between 2013 and 2024. The analysis utilised materials from the Department of Forensic Medicine at the Medical University of Bialystok in Poland. This article builds on an earlier study conducted by the Department in 2010–2011 regarding the prevalence of medicines and psychoactive substances among drivers [13].

## 2. Materials and Methods

Our study is an analysis of materials gathered by the Department of Forensic Medicine at the Medical University of Bialystok in Poland. The dataset comprises toxicology reports from drivers who survived accidents, as well as those who were stopped during roadside surveys in the Podlasie Province and neighbouring areas. Furthermore, it includes toxicology records of drivers involved in fatal traffic accidents who underwent medico-legal autopsies. Authorities ordered blood sample analysis at the Department of Forensic Medicine, Medical University of Bialystok.

The results of our analysis were also presented at the 20th Congress of the Polish Society of Forensic Medicine and Criminology, the 41st Conference of Forensic Toxicologists, and the 9th Symposium of Forensic Geneticists. Bydgoszcz, Poland, 10–12 September 2025 [14].

The Department of Forensic Medicine collected a total of 266 blood samples from 2013 to 2024. Over these twelve years, the distribution of sample counts each year was as follows: 2013 (47 samples), 2014 (89 samples), 2015 (11 samples), 2016 (13 samples), 2017 (22 samples), 2018 (18 samples), 2019 (17 samples), 2020 (12 samples), 2021 (10 samples), 2022 (9 samples), 2023 (10 samples), and 2024 (8 samples). During this period, 79 samples were taken from drivers who died at the scene of the accident. Among these, 51 samples were obtained through medico-legal autopsies, while 28 were collected on-site and sent to the Department. Additionally, there were 61 samples from drivers who survived the accident. Furthermore, 126 samples were taken from drivers during routine roadside surveys.

All samples were tested promptly following the medico-legal autopsy or immediately upon being sent to the Department at the request of the Prosecutor’s Office or Police. In several instances, the requests specifically included the need for blood alcohol content analysis. Samples collected during autopsies conducted at the Department were all examined for blood alcohol concentration. Additionally, 41 samples were submitted to the Department specifically for testing blood alcohol content.

Blood samples were collected using standard vacuum containers that have a valid shelf life. Preliminary toxicology analyses for psychoactive substances were conducted using ELISA immunoenzymatic tests (Neogen R). This procedure aligns with the Regulation by the Polish Minister of Health dated 16 July 2014, pertains to the list of substances with effects similar to alcohol and the conditions and methods for testing their presence in the body. The main groups of substances covered by this regulation, which can be detected using Forensic Drug Detection ELISA Kits, include: opiates; amphetamines; methamphetamine/MDMA; cocaine/BZE; THC; and benzodiazepines. 

In the next phase of the toxicological analysis, a comprehensive screening was performed using gas chromatography coupled with mass spectrometry (GC-MS; Thermo Trace 1310 ISQ LT, Thermo Scientific, Waltham, MA, USA) and liquid chromatography coupled with mass spectrometry (LC-MS; HPLC ACCELA, FINNIGAN LTQ, Thermo Scientific, Waltham, MA, USA). All positive findings from the screening stage were confirmed through LC-MS/MS in Multiple Reaction Monitoring (MRM) mode (Dionex Ultimate 3000, Dionex, Sunnyvale, CA, USA/TSQ Endura, Thermo Scientific, Waltham, MA, USA). Blood alcohol content was determined using headspace gas chromatography with flame ionisation detector (HS-GC-FID; HS Trade GC Ultra; Thermo Scientific, Waltham, MA, USA).

## 3. Results

### 3.1. Gender

Between 2013 and 2024, the study involved 266 drivers, comprising 251 men and 15 women. Among these individuals, 70 men and 9 women lost their lives in fatal traffic accidents. Blood samples were obtained either during the autopsy or collected at the accident scene. The remaining 187 were divided into two groups: those who survived non-fatal accidents and those stopped by police for suspicious driving behaviour, such as rapid acceleration or deceleration, failing to signal, or driving at inappropriate speeds. Blood samples were collected from all non-fatal accident survivors and drivers stopped for suspicious behaviour.

### 3.2. Distribution of Positive Samples

Of the 79 drivers who died in accidents, alcohol or other psychoactive substances were detected in 45 cases, accounting for 57% of this group. Similarly, in the group of 61 individuals who survived accidents, 22 tested positive for intoxicants, representing 36.1%. Among 126 drivers stopped by law enforcement for suspicious driving, 85 had positive test results, accounting for 67.5% of that group (see Table 1).

### 3.3. Single and Polydrug Combinations

Out of all the analysed samples, 164 individuals, representing 61.7% of the total, tested positive for a psychoactive substance, either with or without alcohol. THC was found alone in 62 cases (37.8%) and in combination with other substances in 14 cases (8.5%). THC alone was most commonly identified among drivers stopped during routine roadside checks, accounting for 53 cases, or 32.3% of all positive results. The substance most often combined with THC was benzodiazepines. Benzodiazepines were found in all studied groups. Overall, benzodiazepines accounted for 4.9% of all confirmed cases.

It is also worth noting that among drivers stopped for roadside checks, 18 tested positive for amphetamine alone, accounting for 11% of all samples analysed.

The largest group among the fatalities consisted of drivers under the influence of alcohol alone. Amphetamine was the substance most commonly combined with alcohol.

The least frequently detected substance was cocaine and its metabolite, benzoylecgonine. Cases involving psychotropic medications, psychotropic medications combined with opioids, alcohol with THC and amphetamine, alcohol with psychotropic drugs and stimulants, alcohol with psychotropic medications, or alcohol with amphetamine and benzoylecgonine were equally rare (see Table 2).

### 3.4. Blood Concentration Ranges of Detected Substances

The analysis of the tested substances’ concentrations was based on the charts by Winek et al. (2001) [15]. Among the individuals examined, alcohol was detected in 44 persons—9 during roadside checks (with the highest concentration recorded at 3.2‰) and 35 among drivers involved in accidents, of whom 30 were fatal cases (the highest concentration found in a deceased driver was 4.1‰). A blood alcohol concentration above 0.2‰ is considered in Poland to be under the influence of alcohol [16], while the lethal dose of alcohol depends on many factors. Still, this number is generally accepted as a concentration above 4‰ [17].

THC was found in 72 individuals, comprising 61 from roadside checks and 11 among those involved in accidents. Five drivers died at the scene, with the highest concentration reaching 20 ng/mL. In 49 cases, the THC level exceeded the effective dose (>2.5 ng/mL) [15].

Amphetamine was also detected—38 drivers tested positive, of whom 15 were involved in road accidents. Ten drivers died at the scene, and in six of these cases, amphetamine concentrations exceeded the effective dose (>110 ng/mL) [15].

In seven accidents, drivers were under the influence of opiates—two of these were fatal, with concentrations of 100 ng/mL fentanyl (therapeutic range < 100 ng/mL) [15] and 15 ng/mL methadone (therapeutic range < 1110 ng/mL) [15].

Cases involving benzodiazepines were also identified—four during roadside checks and eight among accident participants, including two fatalities. The most frequently detected substances in this group were diazepam, midazolam, and clonazepam. For all individuals with detected diazepam, the concentrations were within the therapeutic range, below 5 µg/mL [15]. In one case, the concentration of midazolam exceeded the therapeutic level (<25 ng/mL) [15]. In contrast, the level of clonazepam in all drivers remained within the therapeutic range (<75 ng/mL) [15].

Five drivers under the influence of psychotropic substances caused accidents, four of which were fatal accidents, most commonly after taking carbamazepine. In all cases concentrations were within the therapeutic range.

Three drivers under the influence of cocaine were detected—two involved in accidents and one during a roadside check. Among the accidents, one resulted in the driver’s death at the scene, with a blood concentration of 4 ng/mL. In all cases, the concentration did not exceed the toxic level (<900 ng/mL) [15].

Ten drivers were under the influence of stimulants: PMMA (paramethoxymethamphetamine) or MDMA (3,4-methylenedioxymethamphetamine). Eight of them were involved in accidents, six of which resulted in the driver’s death at the scene.

All blood concentration ranges are listed in Table 3.

### 3.5. Detection of Substances Categorised by Type of Vehicle

Among 175 car drivers, 5 motorcyclists, and 13 operators of other vehicle types, substance use was detected. The most commonly used substance among car drivers and motorcyclists was THC. Among those operating other vehicle types (tractors, bicycles), the most frequently detected substance was alcohol, present in 35.0% of individuals. The second-most common substance among car drivers was alcohol (21.7%). For motorcyclists, the second-most frequently detected substances were opiates (20.0%) and benzodiazepines (20.0%). Among drivers of other vehicle types, the second-most common substance was amphetamine (23.5%). Details are referenced in Table 4.

### 3.6. Substance Detection over Time

Table 5 displays the detection frequency for each substance or group of substances found in drivers over the 12 years (2013–2024). THC was the most frequently detected substance (71 cases), followed by amphetamine (37 cases). Both substances showed a declining trend throughout the study period. In contrast, the detection of psychotropic drugs experienced a slight increase, particularly from 2017 onwards. Benzodiazepines showed a slight decrease over time. The prevalence of other substances, such as opiates, cocaine, and stimulants, remained relatively stable, with no significant changes observed across the years. Detection of alcohol was consistently higher than that of other groups; however, its frequency also remained steady over time.

### 3.7. Single and Polydrug Samples

**Figure 1:**
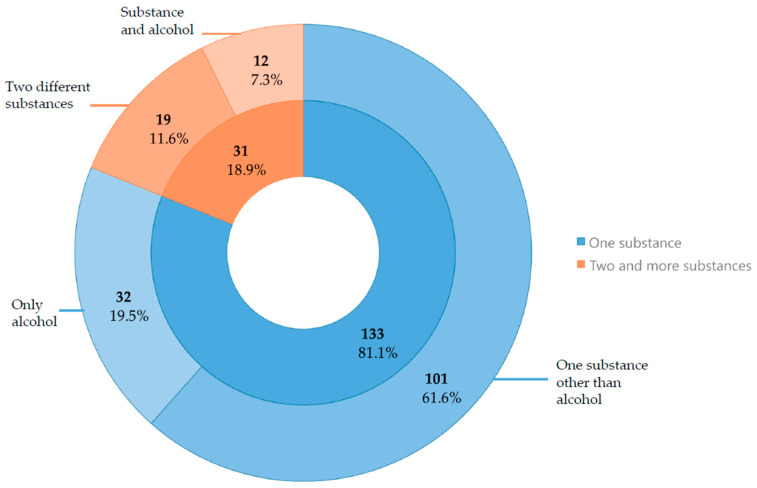
Number and percentage of substances used alone versus mixed, with a distinction for alcohol.

Figure 1 displays the number and percentage of drivers under the influence of various substances. The analysis categorises drivers into two groups: those who used a single substance and those who used multiple substances. The first group includes drivers who used only one substance (133 cases), further divided into those who consumed only alcohol (32 cases) and those who used substances other than alcohol (101 cases). Most drivers had used only one substance. The second group consists of drivers who used two or more substances (31 cases), split into those who consumed alcohol alongside other substances (12 cases) and those who used multiple substances excluding alcohol (19 cases).

## 4. Discussion

The ongoing development of transportation methods has resulted in a more diverse population of drivers on the roads. At the same time, increased accessibility and a wider variety of newer medications, psychoactive substances, and alcohol create a potentially dangerous combination. Today, many individuals consume substances that can impair their ability to drive. These substances can be classified as either depressants—such as alcohol, benzodiazepines, opioids, THC—or stimulants like amphetamines, MDMA, PMMA, and cocaine. Depressants significantly impair reaction times, reduce concentration, cause dizziness, and hinder information processing [18,19,20,21,22]. In contrast, stimulants may lead to risky behaviours, aggressive driving, and difficulty in maintaining attention and focus. They can also cause hallucinations, disordered thinking, visual disturbances, and impaired coordination [23,24,25]. The concurrent use of multiple psychoactive substances markedly increases the likelihood and severity of these effects. Driving under such influence greatly heightens the risk of accidents and dangerous behaviour on the road [26].

According to our findings, THC was the most common substance detected in drivers—37.8% of positive blood samples. This aligns with recent studies from Denmark (2015–2016) [27], Italy (2014–2017) [28], and Hungary (2014–2015) [29], where THC was also the most frequently identified psychoactive substance aside from alcohol. Alcohol was next at 19.5% of samples. The number of blood alcohol-positive samples—32—may seem low considering that 2616 people in Podlasie Province were caught driving under the influence in 2024 [30]. This discrepancy can be attributed to the recent use of private laboratories for sobriety tests and the study’s focus on cases where authorities requested toxicology reports.

Amphetamine was the third-most commonly detected substance, accounting for 12.8% of samples, similar to previous findings where it followed THC [13]. Notably, opiate detection decreased from 15.9% to 2.4% [13]. An essential addition is a new category of drugs—stimulants established as synthetic substances, such as PMMA, alpha-PVP, and MDMA, which have become more popular on the Polish market. Known as legal highs, these psychoactive substances were first introduced around 2008, when the first specialised legal high shops began to appear on Polish streets. This phenomenon reached its peak in 2010, with over 1300 such shops in existence [31]. Along with the popularity of these substances among adolescents and school students, this led to legal changes that contributed to a decline in stimulant use. A steady decrease has been observed since 2011 [32]. Our analysis does not mirror the Polish trend, as stimulant use among the drivers we analysed peaked in 2017 and again in 2020–2021. Nonetheless, our results can be linked to an increase in the detection of new synthetic stimulants on the Polish market, which reached an all-time high in 2016, then decreased for some time before rising again in 2021 [32].

In our study, cocaine is overtaken by several other types of drugs. In Italy and Denmark, it ranks as the second-most commonly detected substance among drivers, at 7.2% [28] and between 27% and 28.5% [27] of all detections, respectively. Our results are consistent with European trends in psychoactive substance use. The European Union Drugs Agency report on cocaine states that the prevalence of cocaine in Poland is among the lowest in European countries [33].

Considering multidrug use, THC was used as often as amphetamine, with both appearing in combinations in 13 cases. Previously, amphetamine was combined with other substances in only 9 cases [13]. Alcohol was frequently mixed with stimulants, including amphetamine, in 6 cases. An Italian study reached a different conclusion, finding that in the Milan area, alcohol was most commonly paired with cocaine and cannabinoids [34]. Similar results to ours were observed by Gjerde et al. in Norway, where THC was the most frequently detected drug used in combination with other substances [35]. The most common substance combinations observed were THC with sedatives and stimulants (22.9%), THC with alcohol (14.1%), THC with sedatives alone (14.1%), and the combination of THC, alcohol, and sedatives (11.9%) [35]. An analysis carried out in the USA showed that the largest group of drivers under the influence was composed of those using alcohol alone (54.9%), followed by a group mixing alcohol with cannabinoids (36.5%) [36].

Maurer et al. over a period of two years (2018–2019) in Western Switzerland, identified one or more psychoactive drugs in 89.0% of cases [37]. In our study, we recorded only a few instances of this, which can be partly attributed to the fewer requests for comprehensive toxicology panels.

In our study, the highest BAC detected was 4.1‰ in a road traffic accident victim, a level considered life-threatening. The highest BAC measured during roadside surveys was 3.2‰. It is impossible to compare this to the previous study because at that time, samples were collected only from sober drivers. Ferrari et al. conducted a similar study in the Milan area, reporting the highest recorded BAC of 4.6‰ [34], and Maurer in Western Switzerland—recording a BAC of 3.8‰ [37].

The highest THC concentration recorded in our study was 20 ng/mL, found in the blood of a victim involved in a road traffic accident. This level exceeds the previously highest reported value of 12.3 ng/mL from a roadside survey, as documented in our previous study [13]. The maximum blood concentration reported by Maurer was 52.0 µg/L [37].

A Norwegian study found that 20.0% of drivers involved in crashes had blood THC levels above 1.3 ng/mL, which is the legal limit in their country. Concentrations exceeding 9.3 ng/mL (high sanction limit) were recorded in 14.8% of cases [35]. Italian research showed that over 75.0% of samples testing positive for THC were above the legal limit of 2 ng/mL [34].

Our analysis included blood concentrations of various stimulants, such as amphetamine, cocaine, PMMA, MDMA, alpha-PVP, and mephedrone. The highest amphetamine concentration was found in the blood of a road traffic accident victim, at 2997 ng/mL—over 27 times higher than the effective dose of 110 ng/mL. This significantly exceeds our previous research, where the highest recorded concentration was 155.2 ng/mL during a roadside survey [13]. Gjerde et al. documented almost 6.0% of cases where blood concentrations of amphetamine exceeded 487 ng/mL [35]. A different study conducted in Norway, published in 2024, recorded a median blood amphetamine concentration of 299.7 ng/mL [38], and a Swiss study, 1700.0 μg/mL [37].

Cocaine was detected at its peak blood concentration of 30.0 ng/mL during a roadside survey, while its metabolite, benzoylecgonine, reached 602.0 ng/mL in the blood of an accident survivor. Our previous work focused on the blood concentration of benzoylecgonine rather than cocaine itself, reporting a maximum concentration of 117.3 ng/mL [13]. In Switzerland, the highest recorded concentration of cocaine was 1500.0 µg/L, and benzoylecgonine 5400.0 µg/L [37].

The remaining substances are not reflected in our earlier work due to their relatively late introduction to the market and their growing popularity over time. PMMA’s highest blood concentration, 360.0 ng/mL, was found in a sample from a road traffic victim; otherwise, the highest blood concentration of MDMA (75.0 ng/mL) was obtained from a roadside survey. MDMA was also detected in a Swiss study, reaching a blood concentration of 1400.0 µg/L [37]. The highest blood concentrations of other stimulants were identified in blood samples from road traffic accident survivors: mephedrone (37.5 ng/mL) and alpha-PVP (120.0 ng/mL).

In this study, we measured blood concentrations of detected drugs and compared them to established therapeutic ranges and known toxic doses. The first group of prescription medications analysed comprised opiates. Notably, opiate concentrations in all cases remained within therapeutic ranges, and opiates were identified exclusively in individuals involved in road traffic collisions. This finding contrasts with our previous study, which observed similar blood concentrations of opiates among all examined groups of drivers. The second group included benzodiazepines, where only one sample exceeded the therapeutic range—midazolam at a concentration of 1200.0 ng/mL, detected in a sample from a survivor of a road traffic accident. Past research did not reveal any cases of benzodiazepine concentrations over the therapeutic range. A similar result was obtained by Maurer et al., with many instances exceeding the therapeutic range [15,37].

Additionally, several psychotropic medications were found in the analysed blood samples; however, all were within their therapeutic ranges, consistent with our previous findings [13].

In our paper, we chose to compare substance use among drivers of different vehicles, mainly between car drivers and motorcycle drivers. Due to the small number of non-car drivers, it is challenging to draw definitive conclusions. However, a Serbian study showed that nearly 23.0% of motorcycle drivers are under the influence, whereas only 0.8% of car drivers were under the influence [39].

We sorted our data to identify trends in substance abuse among drivers over the years. However, due to the small number of cases, most results are inconclusive. A notable decline in THC detections occurred after 2015, likely because authorities in Poland now use private laboratories. Since then, the number of positive samples has stabilised. This does not align with the national decline in THC use, observed since 2011 [31]. Our results also do not reflect national amphetamine trends. Usage among Polish citizens dropped rapidly between 2010 and 2014, then gradually increased [33], unlike our findings. General substance statistics may not directly mirror usage among drivers, but they remain relevant because government data often lacks a focus on driving under the influence. Statistics mainly emphasise alcohol-related driving. Alcohol consumption in the general population remained steady, apart from a slight increase in spirit-based drinks [32]. Except for 2017, our alcohol analysis produced consistent results. In contrast to the overall trend in Europe, alcohol-related driving did not decrease universally; a 2016 study in Campania, Italy, found a significant rise over eight years [40].

In our research, we chose to emphasise alcohol use; however, the small number of alcohol-positive samples limits interpretation. While alcohol is less likely to be used alone or with other substances in our findings, a US study found only 2.0% of respondents reported driving under the influence of multiple substances, though among those who drove under the influence, a significant proportion were polydrug users: 16.0% reported using two substances, and 3.5% reported using three or more [36].

## 5. Conclusions

Although there has been a decline in the detection of psychoactive substances in recent years, drivers under the influence of alcohol and/or drugs still pose a significant threat to road safety.

Among the psychoactive substances most often used alone or alongside others, THC predominated.

It is crucial to implement new and enhanced screening methods during roadside police checks.

It is also necessary to implement prevention strategies such as public awareness campaigns and stricter regulations, including zero-tolerance policies for driving under the influence of psychoactive substances.

Roadside checks were mainly carried out on male drivers, which could be related to risky driving behaviours.

## Figures and Tables

**Table 1:** Distribution of “positive” cases according to the category of drivers.

	Drivers Who Died at the Scene	Drivers Who Survived an Accident	Drivers Stopped for a Roadside Survey
The number of examined blood samples	79	61	126
The number of “positive” blood samples	45	22	85
The percentage of “positive” blood samples	57.0%	36.1%	67.5%

**Table 2:** The number and prevalence of medicines and psychoactive substances in the blood samples of drivers.

	Drivers Who Died at the Scene	Drivers Who Survived an Accident	Drivers Stopped for a Roadside Survey	The Number of “Confirmed” Blood Samples	The Percentage of “Confirmed” Blood Samples
THC	4	5	53	62	37.8%
THC + Benzodiazepines	0	4	1	5	3.1%
THC + Opiates	0	0	0	0	0.0%
THC + Opiates + Benzodiazepines	0	0	0	0	0.0%
THC + Amphetamines	0	0	4	4	2.4%
THC + Cocaine + Benzoylecgonine	0	0	1	1	0.6%
Cocaine + Benzoylecgonine	1	0	0	1	0.6%
Amphetamine	3	0	18	21	12.8%
Amphetamine + Opiates	0	0	0	0	0.0%
Amphetamine + Benzodiazepines	0	0	0	0	0.0%
Opiates	1	3	0	4	2.4%
Benzodiazepines	2	3	3	8	4.9%
Stimulants	1	1	1	3	1.8%
Stimulants + Amphetamine	1	0	1	2	1.2%
Opiates + Benzodiazepines	0	2	0	2	1.2%
Stimulants + Cocaine	0	0	0	0	0.0%
Stimulants + Psychotropic	0	0	0	0	0.0%
Psychotropic	1	0	0	1	0.6%
Psychotropic + Stimulants + Cocaine	0	1	0	1	0.6%
Psychotropic + Opiates	1	0	0	1	0.6%
Other	0	0	0	0	0.0%
Alcohol	20	3	9	32	19.5%
Alcohol + THC + Amphetamine	1	0	0	1	0.6%
Alcohol + Amphetamine	3	1	0	4	2.4%
Alcohol + Amphetamine + Stimulants	1	0	0	1	0.6%
Alcohol + Benzoylecgonine	0	0	0	0	0.0%
Alcohol + Benzodiazepines	0	1	0	1	0.6%
Alcohol + Psychotropic + Stimulants	1	0	0	1	0.6%
Alcohol + Psychotropic	1	0	0	1	0.6%
Alcohol + Stimulants	2	0	0	2	1.2%
Alcohol + Others	0	0	0	0	0.0%
Alcohol + Amphetamine + Benzoylecgonine	1	0	0	1	0.6%
Acetone + THC	0	1	1	2	1.2%
THC + Benzoylecgonine	0	0	1	1	0.6%
Benzoylecgonine	0	0	1	1	0.6%
Total	45	25	94	164	100%

**Table 3:** Concentration ranges of substances detected in drivers involved in accidents and subjected to inspection.

Substance	Road Accidents		Drivers Stopped for a Roadside Survey
	Drivers Who Died at the Scene	Drivers Who Survived the Accident	
	Concentration in Blood ng/mL; Blood Alcohol Content in ‰	Concentration in Blood ng/mL; Blood Alcohol Content in ‰	Concentration in Blood ng/mL; Blood Alcohol Content in ‰
THC	1.2–20.0	0.2–4.2	1.4–4.5
Amphetamine	15.0–2997.0	41.0–214.0	19.3–1463.0
Diazepam	55.0–250.0	300.0	170.0–480.0
Midazolam	0	17.0–1200.0	0
Clonazepam	0	21.0	22.0–36.0
Tramadol	15.0	70.0–600.0	0
Fentanyl	100.0	45.0	0
Morphine	0	66.0	0
Methadone	0	174.0	0
Cocaine	4.0	10.0	30.0
Benzoylecgonine	38.0–260.0	602.0	59.0–413.0
Carbamazepine	8.0–2100.0	0	0
Zolpidem	0	233.0	0
Citalopram	320.0	0	0
Opipramol	220.0	0	0
PMMA	45.0–360.0	0	0
MDMA	20.0	0	75.0
Mephedrone	0	37.5	0
alfa-PVP	0	120.0	0
Alcohol	0.1–4.1	1.3–3.4	0.2–3.2

**Table 4:** Detection of substances categorised by type of vehicle.

Substance	Car	Car%	Motorcycle	Motorcycle%	Other	Other%
Alcohol	38	21.7%	0	0%	6	35%
THC	66	37.7%	3	60%	3	17.7%
Amphetamine	34	19.4%	0	0%	4	23.5%
Opiates	6	3.4%	1	20%	0	0%
Benzodiazepines	9	5.1%	1	20%	2	11.8%
Psychotropic	4	2.3%	0	0%	1	5.9%
Cocaine	3	1.7%	0	0%	0	0%
Benzoylecgonine	6	3.4%	0	0%	0	0%
Stimulants	9	5.1%	0	0%	1	5.9%
Total	175	100%	5	100%	17	100%

**Table 5:** Detection of each substance or substance group over the years.

Year	THC	Amphetamine	Opiates	Benzodiazepines	Psychotropic	Cocaine	Benzoylecgonine	Stimulants	Alcohol
2013	15	7	1	1	0	0	1	0	7
2014	36	10	1	4	0	0	0	0	4
2015	0	1	0	1	0	0	0	0	1
2016	3	0	0	0	0	0	1	1	2
2017	3	4	0	0	1	1	1	4	9
2018	1	3	2	3	1	0	0	0	4
2019	2	3	1	0	0	0	1	1	4
2020	3	3	0	0	0	0	0	2	4
2021	3	3	0	1	0	0	0	2	2
2022	4	3	0	0	1	1	1	1	0
2023	1	0	1	1	0	1	1	0	4
2024	0	0	1	0	2	0	0	0	3

## Data Availability

The data generated by this research are all presented in the article.
